# Evidence-Based Practice: a survey regarding behavior, knowledge, skills, resources, opinions and perceived barriers of Brazilian physical therapists from São Paulo state

**DOI:** 10.1590/bjpt-rbf.2014.0102

**Published:** 2015-09-01

**Authors:** Tatiane M. Silva, Lucíola C. M. Costa, Leonardo O. P. Costa

**Affiliations:** 1 Programa de Mestrado e Doutorado em Fisioterapia, Universidade Cidade de São Paulo (UNICID), São Paulo, SP, Brasil; 2 Musculoskeletal Division, The George Institute for Global Health, Sydney, NSW, Australia

**Keywords:** Evidence-Based Practice, physical therapy, cross-sectional studies, Brazil

## Abstract

**BACKGROUND:**

Evidence-Based Practice (EBP) has been widely used by health professionals. However, no study in Brazil has investigated the data regarding the knowledge and difficulties related to EBP from a representative sample of physical therapists.

**OBJECTIVE:**

To identify behavior, knowledge, skills, resources, opinions and perceived barriers of Brazilian physical therapists from the state of São Paulo regarding EBP.

**METHOD:**

A customized questionnaire about behavior, knowledge, skills, resources, opinions and perceived barriers regarding EBP was sent by email to a sample of 490 physical therapists registered by the Registration Board of São Paulo, Brazil. Physical therapists who did not respond to the questionnaire were contacted by telephone and/or letter. The data were analyzed descriptively.

**RESULTS:**

The final response rate was 64.4% (316/490). Because 60 physical therapists were no longer practicing, 256 answers were analyzed. The physical therapists reported that they routinely read scientific papers (89.5%) as a resource for professional development, followed by continuing education courses (88.3%) and books (86.3%). Approximately 35% of the respondents reported a clear understanding of the implementation of research findings in their practice; approximately 37% reported no difficulties in critically appraising scientific papers; and 67.2% strongly agreed that EBP is important for their practice. The most commonly reported barriers were related to difficulties in obtaining full-text papers (80.1%), using EBP may represent higher cost (80.1%) and the language of publication of the papers (70.3%).

**CONCLUSION:**

Physical therapists from São Paulo state believe that they have knowledge and skills to use EBP. Although they have favorable opinions regarding its implementation, they still encounter difficulties in implementing EBP successfully.

## Introduction

Evidence-Based Practice (EBP) is defined as "the conscientious, explicit, and judicious use of current best evidence in making decisions about the care of individual patients"^1^. However, patient's expectations, wishes and values, as well as the experience of the professional practitioner, also needs to be considered in the decision-making process^2^. EBP has been used as an important decision-making model^3^ and contains five basic steps that should be followed to achieve success in applying its principles: 1) formulation of a clinical question; 2) conduct of an efficient database search to answer the clinical question; 3) critical assessment of the validity of the evidence; 4) application of the evidence findings in clinical practice; and 5) assessment of the clinical practice effects of the evidence application^4^.

Despite its well-defined principles, some obstacles may interfere in EBP, such as the limited availability of resources, the physical therapist's ability to competently apply an intervention considered to be the best based on the clinical evidence, socioeconomic and cultural factors^2^, or perhaps problems related to current health policies, the complexity of the physical therapy practice, access to full-text papers and continuing education programs^5^.

A recently published systematic review^6^ noted that the main barriers to EBP implementation by physical therapists were: lack of time, inability to comprehend statistical data, lack of employer support, lack of resources, lack of interest and lack of generalization of results of the studies to the patient^6^. The publication language, mostly English, might also be considered a barrier that hinders the use of the pertinent studies due to the lack of understanding by readers who do not speak the language^3^. As an example regarding Brazilian physical therapists, less than 1% of all the applied clinical research indexed in PEDro database (Physiotherapy Evidence Database) is published in Portuguese^7^.

Previous studies have investigated different aspects of EBP in specific physical therapy populations^6^. One pilot study^8^ (n=67) conducted in Brazil has addressed some of these characteristics by physical therapists in the state of Santa Catarina. Therefore, the examination of a representative sample that comprises most of the characteristics that affect EBP implementation by Brazilian physical therapists is needed.

The Health System in Brazil and the training of Brazilian physical therapists have unique characteristics compared with other countries (for example, while educational training in Brazil is performed on a University basis with a 4 to 5 years program, European countries such as the Netherlands and France provide training in a 2-3 years based on a technical program); therefore, a specific investigation is necessary. The present study aimed to identify the behavior, knowledge, skills, resources, opinions and perceived barriers of Brazilian physical therapists from the state of São Paulo (SP) regarding EBP.

## Method

### Study design

This was a cross-sectional descriptive study conducted upon approval by the Ethics Committee of *Universidade Cidade de São Paulo* (UNICID), São Paulo, SP, Brazil, approved on March 20, 2013 (CAAE 13479213.6.0000.0064).

### Participants

This study received institutional support from the Physical Therapy Registration Board of São Paulo (CREFITO-3). This Registration Board provided the data of 490 individuals chosen by random selection. All were physical therapists with valid certification by the Physical Therapy Registration Board of São Paulo, Brazil up to December 2012 and had valid email addresses. CREFITO-3 aided in sending the emails and, later in the research, provided the individual telephone numbers and addresses. All data were confidentially analyzed, with no interference from this Registration Board.

The study's sample size was based on an estimated response rate of 50%. After several simulations using different samples sizes and maintaining the response rate at 50%, it was decided that 450 participants would be needed to achieve high statistical precision. A sample of 450 participants and a 50% response rate, that would represent 225 expected respondents, would provide a sufficiently stable confidence interval that would not significantly change with doubling or quadrupling the sample. To prevent excessive drop out rates, the authors decided to recruit and collect the data from 490 individuals. These calculations were performed with the Confidence Interval Calculator of PEDro database^9^.

### Questionnaire

There was no existing appropriate questionnaire that addressed all the information the authors hoped to collect regarding the analyzed population, so a new questionnaire was developed. The questionnaire (Appendix 1S^[1])^ was developed from questions based on previous EBP studies^10^^-^ 15. This questionnaire consisted of questions divided into eight sections: 1) consent form; 2) current practice status; 3) demographic data; 4) behavior; 5) previous knowledge of EBP resources; 6) skills and available resources; 7) opinions about EBP; and 8) perceived barriers to EBP. The questionnaire was developed with multiple choice answers, and sections five, six and seven contained a 5-point *Likert-*type scale (where 1=strongly disagree, 2=partially disagree, 3=neutral, 4=partially agree, and 5=strongly agree).

To guarantee better quality and understanding of the questionnaire, two pilot studies were performed prior to the final data collection. On the first pilot study, a printed version of the questionnaire was answered and analyzed by 31 physical therapy undergraduate students of UNICID to assess questions comprehension. On the second pilot study the questionnaire was sent via email to 50 physical therapy master's by coursework students of the same university to verify the quality of the submissions and the link-based response process.

### Procedures

The data collection was performed with a formulated questionnaire at the SurveyMonkey website^16^, and was sent via email by CREFITO-3 to all 490 selected physical therapists. The email provided an invitation to participate in the study followed by the link to the questionnaire. All individuals who agreed to participate in the study did so as outlined in the consent terms.

After the questionnaires were provided, the physical therapists had two weeks to answer the questions and to submit their responses. For the physical therapists who did not respond within this period, a new email was sent with the same response timeline. After two weeks, another notification was sent to the non-respondents. A fourth and final notification was sent two weeks later. Subsequently, attempts were made via telephone and then via letter to those with incorrect telephone numbers to maximize the response rate within ethical limits.

### Data analysis

The data were descriptively analyzed using IBM SPSS software, version 19.0 for Windows, and were reported as absolute values, percentages and frequencies.

Based on our results, a secondary analysis was established due to possible differences in the responses according to time from graduation and English-language reading skills, which might be considered predictive of variability in the EBP characteristics evaluated. A gender-based analysis was also performed. These secondary analyses were performed using the chi-square test with the following characteristics: time from graduation, classified into four categories of less than 5 years, 5 to 9 years, 10 to 14 years and more than 14 years; English-language reading skills, classified as poor, moderate, good or excellent; and male or female gender. The level of significance was set at p=0.05.

## Results

**Figure 1.**
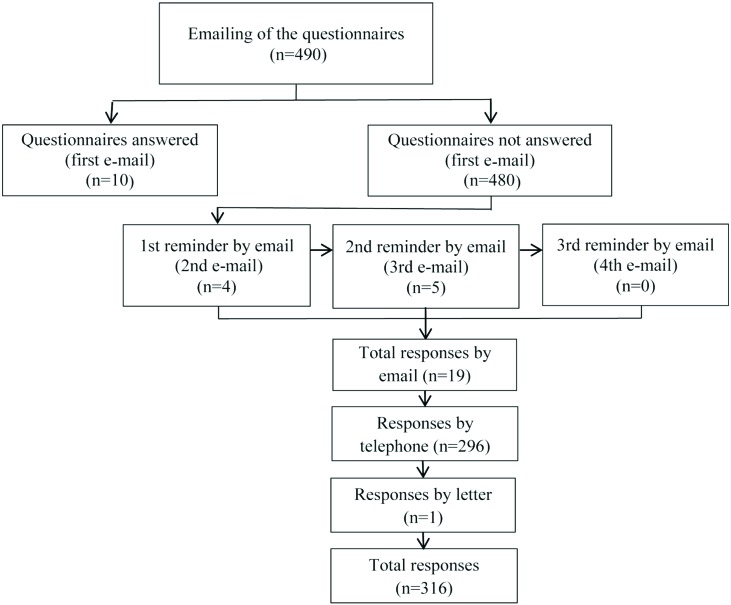
Flow diagram of the study.

The emails were sent by CREFITO-3 to all selected physical therapists. The cumulative response rate for the four email requests was 3.9% (19/490). The response rate from data collected by telephone was 60.4% (296/490 with 22 refusals due to the lack of interest to participate). Letters were mailed to 81 physical therapists who had not responded to the emails and for whom the telephone data were invalid, with only one response. Thus, the final response rate was 64.4% (316/490), which included 256 practicing physical therapists and 60 who were non-practicing. The analyses were conducted with the 256 practicing physical therapists. Figure 1 shows all the phases of the study.

Table 1 describes the demographic characteristics of the participants. The majority of the respondents were female, had graduated less than 5 years prior to the study, held a master's by coursework, had attended private universities, were treating patients and were self-employed. Approximately 55% declared previous experience with research, which probably reflects the final treatises required for graduation and master's by coursework.

**Table 1.** Demographics of the respondents (n=256) of Evidence-Based Practice Questionnaire.

Characteristics	n (%)
Gender	
Female	207 (80.9)
Time from graduation	
Less than 5 years	99 (38.6)
5-9 years	81 (31.6)
10-14 years	45 (17.6)
15-19 years	13 (5.1)
20-24 years	5 (2.0)
More than 24 years	13 (5.1)
Highest level of education	
Bachelor’s degree	78 (30.5)
Master’s by coursework	163 (63.6)
Master’s by research	14 (5.5)
Doctoral	0 (0)
Postdoctoral	1 (0.4)
Type of university/college	
Private	239 (93.4)
Current practice	
Treating patients	248 (96.9)
Teaching	21 (8.2)
Research	16 (6.3)
Other	16 (6.2)
Area of interest	
Musculoskeletal or orthopedics	91 (35.5)
Cardiorespiratory	53 (20.7)
Neurology	35 (13.7)
Dermatology	31 (12.1)
Acupuncture	12 (4.7)
Public health	10 (3.9)
Sports	8 (3.1)
Workplace health	6 (2.3)
Chiropractic and osteopathy	5 (2.0)
Women’s health	4 (1.6)
Urogynecology	1 (0.4)
Oncology	0 (0)
Employment sector	
Self-employed	129 (50.4)
Private	84 (32.8)
Public	32 (12.5)
More than one	11 (4.3)
Previous experience teaching	
No	201 (78.5)
Previous experience with research	
Yes	140 (54.7)
Self reported English-language skills	
Poor	74 (28.9)
Moderate	117 (45.7)
Good	56 (21.9)
Excellent	9 (3.5)

### Behavior in relation to EBP

Table 2 presents the respondents' characteristics regarding their behavior in relation to their use of research resources. The physical therapists reported using scientific papers (89.5%) as a practice resource, followed by courses (88.3%) and books (86.3%). When asked about the databases they had already used, there was a clear preference for a Portuguese/Spanish databases, such as SciELO (86.7%), compared to broader databases, such as PubMed (71.9%) and Cochrane (28.9%), or a physical therapy-specific database, such as PEDro (13.7%).

**Table 2.** Data regarding behavior of respondents and the use of Evidence-Based Practice.

Characteristics	n (%)
Knowledge update methods	
Scientific papers	229 (89.5)
Courses	226 (88.3)
Books	221 (86.3)
Magazine-related articles	191 (74.6)
Meeting, conferences, lectures	174 (68.0)
Study groups	50 (19.5)
Databases used	
SciELO	222 (86.7)
Lilacs	205 (80.1)
Google Scholar	204 (79.7)
PubMed	184 (71.9)
Cochrane	74 (28.9)
PEDro	35 (13.7)
I have never used databases	8 (3.1)
Other	6 (1.2)
Databases more frequently used	
SciELO	122 (47.7)
PubMed	68 (26.6)
Bireme	50 (19.5)
Google Scholar	48 (18.8)
Lilacs	46 (18.0)
I do not use databases	17 (6.6)
PEDro	4 (1.6)
Cochrane	2 (0.8)
Other	1 (0.4)
Frequency of database use	
Everyday	5 (2.0)
1 to 3 times a week	62 (24.2)
1 to 3 times a month	70 (27.3)
Once every 2 months	23 (9.0)
Very rarely	38 (14.8)
I do not use databases	58 (22.7)
Site of database use	
Home	205 (80.1)
Work	64 (25.0)
University	25 (9.8)
Other	1 (0.4)

### Knowledge, skills and resources, and opinions related to EBP

Table 3 shows the percentage of physical therapists' responses by category to questions regarding their knowledge, skills, resources, and opinions related to EBP. The physical therapists reported having a clear understanding regarding the use of research findings in clinical practice (41.8% strongly agreed and 35.5% partially agreed) and about different types of study designs (40.2% strongly agreed and 37.5% partially agreed) and having sufficient knowledge to apply EBP (27.7% strongly agreed and 43.4% partially agreed); however, inconsistency was exhibited in the understanding of the core elements of EBP and about statistical data.

**Table 3.** Knowledge, skills, resources, opinions of physical therapy practitioners regarding Evidence-Based Practice.

	Strongly disagree	Partially disagree	Neutral	Partially agree	Strongly agree
Knowledge					
I know the meaning of the term Evidence-Based Practice (EBP).	2.7	1.2	7.8	34.8	53.5
I had no experience with EBP in my graduate or or postgraduate degree.	37.5	19.5	7.8	19.5	15.6
The knowledge that I possessed during my graduate or postgraduate degree regarding EBP was sufficient.	25.4	30.1	10.9	23.4	10.2
I do not understand the core elements of EBP.	26.6	22.3	21.5	22.3	7.4
I have clear understanding regarding the use of research findings in clinical practice.	3.1	7.0	12.5	35.5	41.8
I have an understanding regarding different types of studies (study designs).	3.1	5.9	13.3	37.5	40.2
I do not have understanding of statistical data.	20.3	28.9	11.3	25.8	13.7
I believe I have sufficient knowledge to implement EBP.	5.1	10.9	12.9	43.4	27.7
I am not interested in furthering my knowledge of EBP.	68.8	14.5	7.8	6.6	2.3
Skills and resources					
I am not able to perform database searches.	34.8	28.9	16.0	15.2	5.1
I am able to critically assess a scientific paper.	4.7	7.0	22.3	36.7	29.3
I routinely access online databases.	4.7	7.8	13.7	29.3	44.5
I do not have incentive to implement EBP in my daily practice.	27.0	11.7	22.7	13.7	25.0
I have computer resources and Internet access at my workplace that facilitate the implementation of EBP.	15.2	6.3	17.6	16.8	44.1
I do not have discussions about EBP at my workplace.	32.4	13.7	19.5	12.9	21.5
I ask my patients about their preferences and I consider them in my decision-making.	3.9	4.7	7.8	33.2	49.6
I inform my patients of their treatment options and involve them in the decision-making.	4.3	6.6	6.3	39.5	43.4
I never try to deploy the best scientific evidence in my clinical practice.	54.3	21.5	14.1	7.4	2.7
Opinions					
EBP is important to my clinical practice.	1.2	0.4	6.3	25.0	67.2
I do not believe that EBP improves patient care in physical therapy.	65.6	23.4	6.3	2.3	2.3
Much of my decision-making regarding the treatment of my patients incorporates EBP.	3.5	8.6	15.6	41.0	31.3
An expert’s opinion in my field is the most important factor in my decision-making process.	5.5	17.2	15.6	44.9	16.8
The use of the best current scientific evidence does not benefit the quality of health services.	48.8	31.3	10.9	7.0	2.0

Variables expressed in percentages.

From the questions regarding skills and resources, it was noted that physical therapists reported no difficulties in critically assessing a scientific paper (29.3% strongly agreed and 36.7% partially agreed) and that they reported routinely accessing databases (44.5% strongly agreed and 29.3% partially agreed).

Regarding the questions about their opinions concerning EBP, the physical therapists reported being in favor of EBP since 67.2% strongly agreed and 25% partially agreed that EBP is important to clinical practice and 65.6% strongly agreed and 23.4% partially agreed that EBP improves patient care. Additionally, 31.3% strongly agreed and 41% partially agreed that EBP importantly contributes to their clinical decision-making; conversely, 16.8% strongly and 44.9% partially agreed that an expert's opinion is the most important factor in decision-making process.

### Barriers to EBP

**Figure 2.**
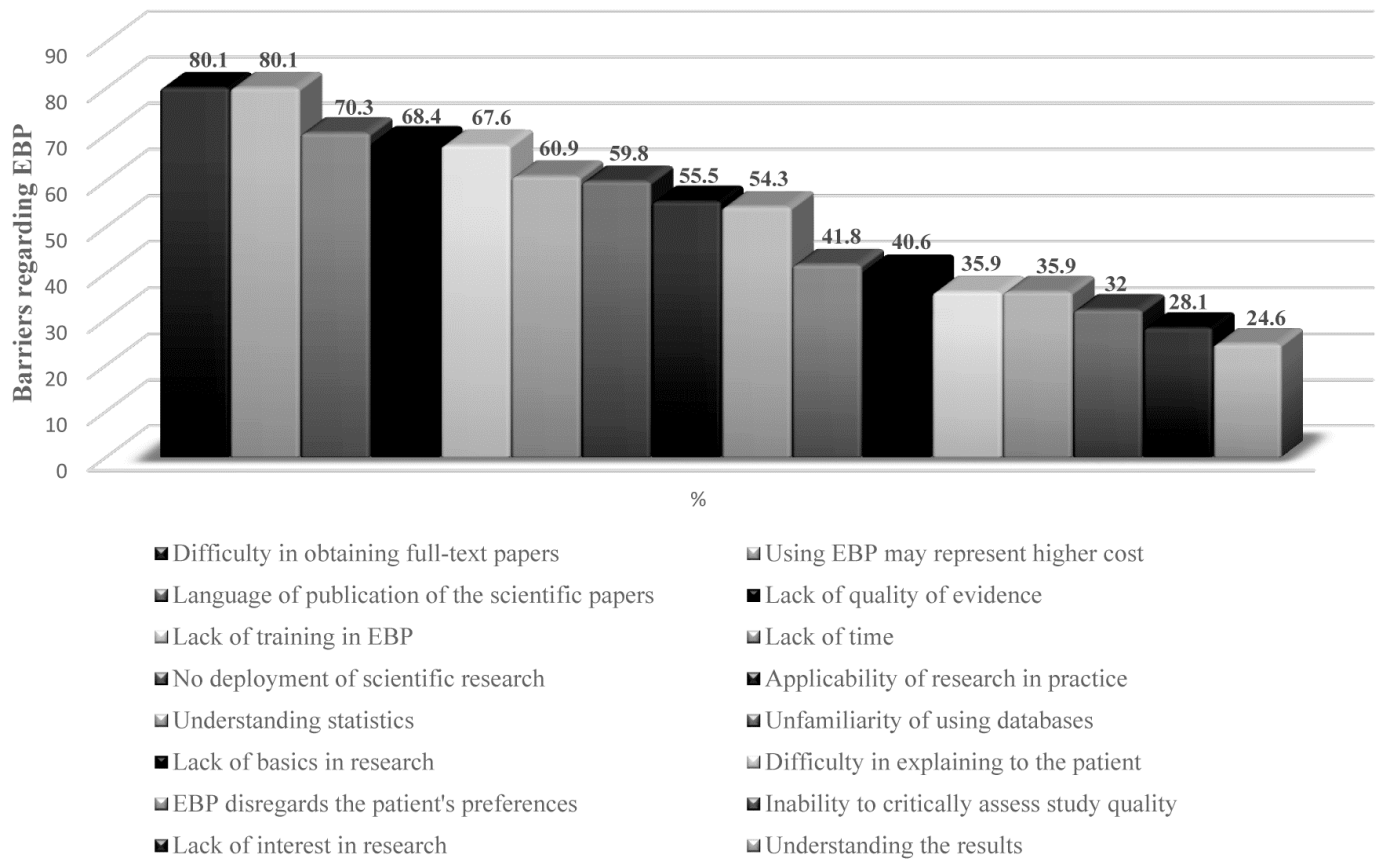
Barriers reported by respondents regarding Evidence-Based Practice (in percentages).

The most frequent barriers reported by physical therapists were mostly related to difficulty in obtaining full-text papers (80.1%), using EBP might represent higher cost (80.1%) and the language of publication of the papers (70.3%). The less frequently cited barriers were lack of interest in research (28.1%) and understanding the results of the studies (24.6%). These data are shown in Figure 2.

The secondary analysis showed significant differences between the time-since-graduation categories, suggesting that physical therapists who had graduated within nine years had more knowledge and skills compared with those who had graduated more than nine years ago in response to the following questions: 'I had no experience with EBP in my graduate or postgraduate degree' (p=0.001); 'The knowledge that I possessed during my graduate or postgraduate degree regarding EBP was sufficient' (p=0.004); 'I do not understand the core elements of EBP' (p=0.004); 'I am able to critically assess a scientific paper' (p=0.005); and 'I routinely access online databases' (p=0.009). There were also significant differences in the English-language reading skills categories, suggesting that physical therapists with good or excellent skills had greater knowledge and skills than physical therapists with poor or moderate skills in response to the following questions: 'I do not understand the core elements of EBP' (p=0.009); 'I am not able to perform database searches' (p=0.003); and 'I am able to critically assess a scientific article' (p=0.035). There were no significant differences between genders.

## Discussion

The present study aimed to identify behavior, knowledge, opinions, skills and resources, and perceived barriers of physical therapists living in SP with regard to EBP. Despite favoring EBP implementation, the physical therapists living in São Paulo appear to value experts' opinions as well as using scientific papers, considering that 88.3% reported taking courses for professional development and 89.5% used scientific papers; moreover, 16.8% strongly and 44.9% partially agreed that an expert's opinion was the most important factor in decision-making, which contradicts one of the central pillars of EBP by which evidence should be provided by high-quality clinical research and not by experts' opinions^17^

This finding may be related to the Brazilian education model, which is modeled on the teacher as the main actor in transferring knowledge to the students. This model may give the health professional the impression that knowledge rests on experts' opinions and not on EBP principles, disregarding the use of information contained in scientific papers as an adjunct in clinical decision-making.

Regarding the routine use of online databases, to which 44.5% strongly and 29.3% partially agreed, the most used was the SciELO database (47.7%) and, to a lesser extent, PEDro (1.6%) and Cochrane (0.8%) databases. This preference might be justified by the languages adopted by SciELO database, i.e., Portuguese and Spanish, and the availability of full-text papers in that database. These can be important aspects for the physical therapists who considered the difficulty in accessing full-text papers (80.1%) and the language of publication of the scientific papers (70.3%) as barriers.

However, these physical therapists disregarded the fact that reading papers only available in the full-text version in this condition might cause them to miss the most important papers, which is known as Full Text On the Net (FUTON) bias^18^^,^ 19. Additionally, the review of papers available exclusively in Portuguese or Spanish may not represent the best evidence available, which clearly shows a high level of language bias^3^^,^ 20. Finally, the frequency of database use among professionals was relatively low, and one-quarter of the respondents did not report using any database.

It is noteworthy that Brazil has broad access to databases through Bireme^21^, which grants free access to the Cochrane^22^ and SciELO^23^ databases. Capes e-journal web portal^24^ grants access only to public universities and a few private schools, along with other web portals that provide access to scientific journals. However, to effectively use these databases, proper training of the physical therapists and English-language reading skills are necessary.

The present study showed that physical therapists living in SP reported knowledge and specific skills for implementing EBP, such as the understanding of the application of research findings to clinical practice, the knowledge of different study designs and critically assess scientific paper. However, some of these professionals recognize that the EBP knowledge acquired during graduation was insufficient and that the lack of EBP training was an important barrier to its implementation (67.6%).

The secondary analysis showed that physical therapists with less time since graduation as a physical therapist presented greater knowledge and skills compared with those with longer intervals since graduation as a physical therapist in some of the questions posed. Moreover, physical therapists with greater English-language reading skills also presented this characteristic when compared with those with poor or moderate reading skills. These data suggest that the training of physical therapists in the last decade might have shifted towards the use of research in clinical practice, with the introduction of specific courses on the subject. This also reinforced the importance of English language proficiency to improve the knowledge and specific skills of EBP.

In this study, the authors intended to obtain a random sampling among all registered physical therapy professionals in São Paulo state. Additionally, many efforts were made to maximize the response rate, as suggested in previous studies^25^^,^ 26, using email with three reminders as well as contact by telephone and letter. The authors achieved a satisfactory response rate (64.2%) considering the response rates of similar studies, which have varied between 20%^27^ and 81%^28^. During the data collection, however, the authors found 81 invalid telephone numbers, and this might have negatively affected the final response rate. Surprisingly, the authors observed a very low response rate via email. It appears that this resource still has met with limited success in research in Brazil, unlike in other countries^28^^-^ 30.

Although the Brazilian educational and health systems have particularities, the present data agree with existing studies reporting that physical therapists from other countries also believe they possess the knowledge to build a clinical question^31^^,^ 32, to search online databases^28^, to develop critical assessments^30^^,^ 32 and that they have common difficulties, such as the low frequency of database use^30^^,^ 32 ^,^ 33 and the inability to comprehend statistical data^29^^,^ 34.

The pilot study^8^ conducted with a sample from the state of Santa Catarina demonstrated more optimistic perspectives of the physical therapists since 75% affirmed having knowledge of EBP, 50% reported previous knowledge about its principles, and 59.7% considered themselves confident in the critical analysis of scientific articles, as well as in searching for relevant scientific articles for their clinical practice. Physical therapists who participated in this study were also in favor of EBP implementation, considering that 48% agreed and 40% strongly agreed that EBP is necessary to physical therapy practice, and 68% reported using EBP in their daily practice^8^. Additionally, this study demonstrated important barriers such as lack of time, lack of generalization of results of the studies to patients, lack of information sources, and inability to apply the study's data to individual patients^8^.

The present study allowed the identification of specific barriers in this population, such as the difficulty in obtaining full-text papers and the language of publication of the papers. Efforts should be made to provide physical therapists easier access to scientific papers and to improve physical therapists' English comprehension because more than 90% of the clinical studies in physical therapy are published in English^20^.

The present study permitted a broad view of how EBP is viewed by physical therapists in SP, demonstrating that these professionals believed in having EBP knowledge and the necessary skills. They have also favorable opinions regarding its implementation. However, they exhibited uncertainty when asked about specific habits such as the use of databases, which points to the conclusion that despite EBP being increasingly discussed and its implementation encouraged, important gaps remain to be addressed.

These results imply the need for specific teaching strategies in EBP for this population that should focus on the main difficulties, such as the use of the major databases, which is the second necessary step for EBP application^4^. Moreover, by understanding EBP, the physical therapist is capable of offering a more effective approach, thus reducing the health costs.

One of the limitations of the present study was that no assessment of these professionals' actual knowledge about EBP was made using a specific tool to assess the effectiveness of their training, such as the Fresno test^35^. The Fresno test was recently adapted to Portuguese by Brazilian investigators^36^; however, this new version was not available when the present study was designed.

It warrants acknowledgement that perhaps the physical therapists living in São Paulo state are not representative of professionals from less economically developed regions of the country. However, it is unlikely that the results of this sampling would not also apply to other major centers, such as the capitals of the southern and southeastern states of Brazil. It would be important to conduct a study to confirm these results in a larger number of Brazilian states. Moreover, the possibility exists that in other states (for example, within the southeastern-southern axis) that do not present a significant number of EBP opinion leaders associated with masters or PhD programs, the EBP knowledge scores could be even less favorable.

The present study investigated behavior, knowledge, skills and resources, opinions and perceived barriers of Brazilian physical therapists living in the state of São Paulo, which allowed the identification of the primary difficulties they encountered in implementing EBP. Therefore, the authors suggest further studies on the effect of EBP-specific training skills that might resolve the weaknesses described in the present study.

## Conclusion

Brazilian physical therapists living in São Paulo state believe that they have EBP knowledge and skills, and they present a favorable opinion regarding its implementation; however, difficulties remain in achieving its successful implementation. The primary barriers found in this study were related to obtaining the full-text articles (80.1%), using EBP might represent higher cost (80.1%) and language of publication of the scientific papers (70.3%).
